# Adjustable Gel Texture of Recovered Crude Agar Induced by Pressurized Hot Water Treatment of *Gelidium sesquipedale* Industry Waste Stream: An RSM Analysis

**DOI:** 10.3390/foods11142081

**Published:** 2022-07-13

**Authors:** Cherif Ibrahima Khalil Diop, Sagrario Beltran, Isabel Jaime, Maria-Teresa Sanz

**Affiliations:** 1 Chemical Engineering Section, Biotechnology and Food Science Department, University of Burgos, Pza. Misael Bañuelos s/n, 09001 Burgos, Spain; beltran@ubu.es (S.B.); tersanz@ubu.es (M.-T.S.); 2 Food Technology Section, Biotechnology and Food Science Department, University of Burgos, Pza. Misael Bañuelos s/n, 09001 Burgos, Spain; ijaime@ubu.es

**Keywords:** agar, *Gelidium sesquipedale* waste stream, food industry, pressurized hot water extraction, RSM, texture

## Abstract

A significant amount of bioactive compound-rich solid waste is released during the industrial phycocolloid-centric extraction of *Gelidium sesquipedale*. The impact of mild pressurized hot water extraction on repurposing this waste for the recovery of agar with an adjustable gel texture is investigated. A two-factor interaction response surface model assessed the influences of the operating temperatures (80 to 130 °C), times (45 and 150 min), pressures (1 to 70 bar), and algae concentrations (3 to 10% (*w:v*)). At a temperature of 100 °C, a pressure of 10.13 bar, a recovery time of 45 min, and a 10% algae concentration, the working parameters were considered ideal (*w:v*). Agar with a hardness of 431.6 g, an adhesiveness of −13.14 g.s^−1^, a springiness of 0.94, a cohesiveness of 0.63, and a gumminess of 274.46 g was produced under these conditions. A combined desirability of 0.78 was obtained for the exposed technology that retrieved gels with a minimum agar yield of 10% and thermal hysteresis between 39 ± 1 and 52 ± 0.5 °C. The fitted design can provide a high techno-commercial value to the agri-food industrial waste stream.

## 1. Introduction

The potential for amplified processing in the food industry has resulted in a global increase in waste stream generation. Food wastes containing exploitable organic compounds are traditionally sent to combustion facilities or landfills. As a result, sustainable waste management strategies consider recycling, reuse, and the recovery of profitable value-added compounds viable options. Emerging technologies could play a substantial role in the green recovery of a range of bioproducts from the Agri-Food waste stream through the concept of biorefining. Biofuels, green chemicals, food ingredients, nutraceuticals, pharmaceutical products, cosmetics, biomaterials, and other 2nd and 3rd generation bioproducts, in particular, have gained high industrial interest.

Agar is a marine phycocolloid found in the cell walls of red algae (Rhodophyta). The agar market is expected to develop at a compound annual growth rate (CAGR) of 4.9% between 2016 and 2025, reaching USD 338.17 million by 2022 [1]. Depending on the harvesting period, agar accounts for roughly 40% of the total algae [2], the remaining part generally is considered waste in the seaweed industry. Agar is a mixture of agarose, a linear polysaccharide formed by the alternate of (1-4)- linked 3,6-anhydro-α-L-galactopyrannose and (1-3)-linked-β-D-galactose units [3] and agaropectin. Agaropectin has the same backbone but is grafted with heterogeneous anionic groups such as sulfate, pyruvate, and glycuronate. Agar forms gels with consistent texture throughout a wide temperature range and is commonly referred to as E406 in the food industry. Its thermos-reversible ability makes it an excellent food-grade texture stabilizer agent, allowing for stable homogeneous dispersion [4]. In addition to its potential as a natural food gelling, thickening agent, and non-food precursor, agar’s high fiber content makes it an ideal probiotic [5,6]. Additionally, the challenge of gelatin replacement for the vegetarian, halal and kosher markets has received more attention in the recent decade [7]. The first hydrocolloid with European registration, agar, is known as “vegetable gelatin”. Due to the emergence of bovine spongiform encephalopathy (“mad cow disease”) in the 1980s [8], hydrocolloids such as starch, modified starch, pectin, carrageenan, and agar are widely studied as gelatin alternatives or substitutes.

In the same trend, the industrial extraction of agar has progressed considerably from a simple freezing process in the 1920s to more modern techniques. Microwave-assisted extraction [9], autoclave extraction, accelerated solvent extraction, ultrasounds coupled with hot water extraction [10], and enzyme-assisted extraction [11] are among the emerging technologies employed. According to Barrio et al. [12], ultrasound tended to shorten the extraction time and allow for a higher agar extraction yield. The latter, on the other hand, was shown to be unaffected by ultrasonic power. Martinez-Sanz et al. [13] found that combining sonication and hot water treatments reduced agar extraction time without affecting extraction yield significantly. Pressurized hot water extraction (PHWE) [14], also known as subcritical water extraction under particular conditions, has rarely been explored [15]. Subjecting water to high pressures and high temperatures enhances its solvation power while significantly lowering its density and dynamic viscosity [13]. From a molecular point of view, the rupture of hydrogen bonds in water at subcritical conditions favors the reduction of its dielectric constant due to thermochemical mechanisms. The self-ionization of water molecules is fostered under these conditions, resulting in the formation of hydronium ions (H_3_O^+^) which confer an auto-hydrolysis potential to the green solvent. Whilst pressurized hot water provides a range of benefits, it also shows certain limitations when it comes to extracting thermolabile bioactive compounds [14]. Controlling PHWE parameters such as the process temperature, pressure, time, and the ratio of algae to water was required to achieve a sustainable extraction process of targeted compounds in the *Gelidium sesquipedale* industrial waste stream according to Diop et al. [15]. On the other hand, food textures are directly linked to the structure and composition of the material. They can strongly be related to the process parameters of the technology used and be modified at both microscopic and macroscopic levels [16]. As a result, response surface methodology (RSM), as a collection of mathematical and statistical tools, can be used to create models and evaluate the statistical impact of process parameters on responses [17]. A two-level, three-variable composite central design (CCD) has been evaluated by Barrio et al. [12] to compare conventional extraction and ultrasound extraction methods of agar. The Box-Behnken design, commonly associated with the response surface methodology, does not contain any points at the extremes of the cubic region due to the combination of factors. Furthermore, due to physical constraints on experimentation, the combination of factors could be costly and impossible to test [18]. These constraints could be related to challenges such as the complete combination of several factors to evaluate multiple responses. The Box-Behnken design can provide pertinent experimental models that are influenced by the chosen range of process factors with a reduced number of experiments [19].

In this work, the variability of the gel texture profile of crude residual agar recovered from the discarded algae industry waste stream using pressurized hot water extraction technology will be assessed. To examine the process-structure interactions, the impact of combining varied PHWE parameters will be evaluated. Textural potentials of the recovered crude agar will be analyzed through response surface methodology. The capacity to modify the texture of the residual crude agar by adjusting the correlation between the PHWE process and the hydrocolloid texture while valorizing the algae industry waste stream is a considerable novelty of this study.

## 2. Materials and Methods

### 2.1. Materials

Pressurized hot double distilled water was used as a green solvent to recover the remaining crude agar in the discarded algae waste stream. Hispanagar (Burgos, Spain, https://www.hispanagar.com (accessed on 19 May 2022) generously provided the *Gelidium sesquipedale* waste stream from a primary industrial phycocolloid-centric extraction. The biomass was then rinsed, hand-pressed, and stored at −20 ± 2 °C until use. The algae were then defrosted prior to extraction to obtain a wet biomass containing roughly 75 ± 3% moisture.

### 2.2. Analytical Methods

#### 2.2.1. Extraction of Residual Agar from the Algae Waste

**Figure 1:**
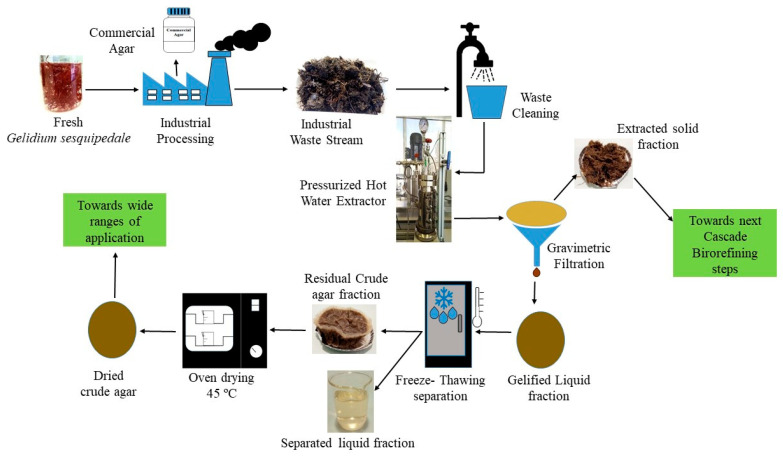
Flow diagram process of the recovery of crude agar from the *Gelidium sesquipedale* industry waste stream using pressurized hot water extraction (PHWE).

Figure 1 summarizes the process of recovering the remaining agar using a 500 mL discontinuous laboratory-designed stainless-steel pressurized liquid extractor vessel as described elsewhere [15]. The corresponding volume of double-distilled water was mixed with the relevant dry weight of the never-dried algae waste stream in the extractor to adjust the solid-to-liquid ratio from 3 to 10% (*w:v*). the injection of N_2_ gas in a static mode (while the outlet valves are closed) was used to control the pressure in the sealed vessel. The reaction system was heated at a rate of 3 °C/min, using an attached heating jacket (230 V, 4000 W, φ95 cm, 160 mm height). When the recovery period was completed, the extractor was depressurized and unsealed after cooling the system to 80 ± 5 °C. A double layer of muslin cloth was used to remove the remaining extracted algae from the hot mixture. The liquid fraction was allowed to gel completely at room temperature. To ensure efficient separation of the agar and the liquid fraction, a two-cycle freeze-thawing (freezing at −20 ± 2 °C for 16 h and then thawing at room temperature) was carried out. The extracted agar was oven-dried at 45 ± 2 °C for 72 ± 5 h or until a constant weight was reached. The three levels of operating factors used to create the Box-Behnken design, including the combination, severity factor, recovery yield, and textural response values of the recovered agar gel, are summarized in Table 1. The PHWE was operated at combined temperatures (from 80 to 130 °C) and pressures (from 1 to 70 bar) according to a previous study [15].

The yield of the recovered crude agar was determined based on the following equation and reported as the percentage of dry mass.

(1) $$\mathrm{Agar}\;\mathrm{yield}=\left(\frac{\mathrm{Dry}\;\mathrm{weight}\;\mathrm{of}\;\mathrm{agar}\;\left(g\right)}{\mathrm{Dry}\;\mathrm{weight}\;\mathrm{of}\;\mathrm{the}\;\mathrm{never}\;\mathrm{dried}\;\mathrm{algae}\;\mathrm{waste}}\right)\times 100$$

#### 2.2.2. Determination of the Physical Properties of the Recovered Agar

FT-IR analysis of the recovered crude agar was performed using a diamond micro-ATR crystal (Jasco FT-IR 4200). IR spectra were captured in 64 scans in the wavenumber range of 400–4000 cm^−1^, which were then processed using Jasco Spectra Manager software (*Spectra Manager*, version 2; JASCO Corporation: Easton, Maryland (EE.UU.), 2002–2012).

The determination of the agar’s gel strength, and the melting and gelling temperature used in the assessment of the hysteresis were also described by Diop et al. [15].

Because the agar’s sol-gel transition temperatures are substantially lower than the gel-sol transition temperatures, the temperature difference between melting and gelling temperatures, also known as thermal hysteresis, was calculated as follows (Equation (2)):

(2) Hysteresis=Melting temperature (°C)−Gelling temperature (°C)

#### 2.2.3. Texture Profile Analysis (TPA)

**Figure 2:**
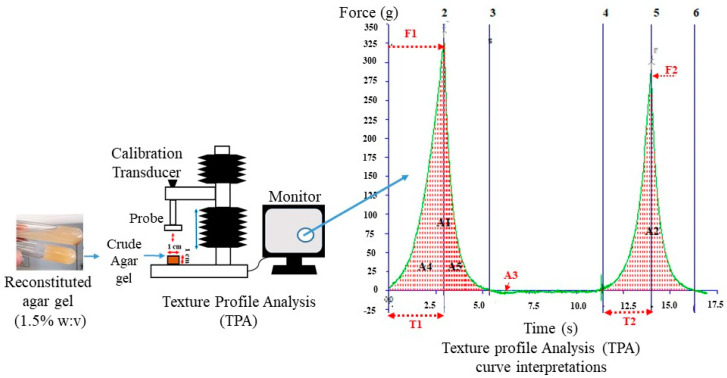
Diagram of the texture profile analysis set, and related double compression curves used in the determination of the texture parameters of the recovered residual agar gel from the algae industry waste stream.

The TPA tests summarized in Figure 2 were carried out using a texture analyzer (Stable Micro Systems model TA-XT2, Surrey, England) equipped with a 50 mm cylindrical diameter probe. The agar gel was molded into a 1 cm × 1 cm (height × diameter) specimen in a cylindrical plastic tube. The test speed was set up at 1 mm·s^−1^, while the pre-test and post-test speeds were both set to 1.5 mm·s^−1^ based on a previously unpublished study. The two-cycle compression process was set to a target distance configured at 30% strain with a trigger force of 5 g.

The data acquisition rate was configured to automatically generate 200 points per second, resulting in the graph of force versus time. All tests were performed in triplicate at room temperature (≈25 °C) with a 5 s delay between the first and second compression.

The load and displacement at specified points on the TPA curves (Figure 2) were used to determine hardness (g), springiness, cohesiveness, adhesiveness (g.s^−1^), and gumminess (g), which were calculated at the time of the test.

The maximal force (F) in grams (g) applied to the gel during the first compression is defined as hardness, which is the force required to indent the material to a certain depth [20]. The negative area under the curve (A_3_) after the sample was subjected to pressure deformation corresponds to the adhesiveness (g.s^−1^), commonly known as sticky mouthfeel in the food sector. It is produced when the sample’s surface adheres to the probe’s surface.

The ratio of time recorded between (i) the start of the second area (*A*_2_) and the second probe reversal (*T_2_*) to (ii) the time (*T_1_*) recorded between the start of the first area (*A*_1_) and the first probe reversal were used to calculate the springiness (Equation (3)).

(3) $$\mathrm{Springiness}=T_{2}/T_{1},$$

The ratio between the positive force areas of the second compression to the first compression is known as cohesiveness (Equation (4))

(4) $$\mathrm{Cohesiveness}=A_{2}/A_{1},$$

where *A*_2_ and *A*_1_ are the respective areas of the second and the first compression curve (Figure 2).

Gumminess is a characteristic of semi-solids, which generally show low hardness and high cohesiveness (Equation (5)):

(5) $$\mathrm{Gumminess}=F\times A_{2}/A_{1},$$

#### 2.2.4. Statistical Analysis

##### Modeling and Experimental Results

During the pressurized hot water extraction process, the temperature (A), internal pressure (B), agar recovery time (C), and algae-to-water ratio (D) were the major process parameters investigated. These operating factors were set at three levels (−1, 0, and 1) (Table 1) in relation to previously published work [15]. The design generated a total of 27 runs with three replicates at the center points.

The pressure in the vessel under experimental conditions carried out without the injection of nitrogen as a pressurized agent was in the range of 1–2 bar depending on the operating temperature. It was equivalent to the vapor pressure; as a result, the first pressure level of the Box-Behnken design was set at 1 bar (Table 1).

A summary statistic test, a lack of fit test, and a sequential sum of squares test were used to assess the suitability of the response surface analysis approach. The model with the highest non-aliased polynomial order and significant additional terms as well as an insignificant lack of fit was chosen.

An ANOVA test was also used to examine the model’s significance, as well as the significance of the individual factors and their two-way interactions, at a 95% confidence level (*p* < 0.05). Multiple non-linear regressions were used to create a second-order quadratic polynomial equation model that correlates the different responses with the key PHWE process parameters. Equation (6) describes the quadratic response by integrating the linear terms, the squared terms, and the linear-by-linear interactions.

(6) $$Y=\beta_{0}+\sum \beta_{i}x_{i}+\sum \beta_{ii}x_{ii}^{2}+\sum \beta_{ij}x_{i}x_{j},$$

where Y is the predicted response surface function, $\beta_{0}$ is the model constant, $\beta_{i}$ is the slope or linear effect of the input factor $x_{i}$, $\beta_{ii}$ is the quadratic effect of input factor $x_{i}$, and $\beta_{ij}$ is the linear by a linear interaction effect between the input factor $x_{i}$ and factor $x_{j}$ [21].

The resultant equation can be simplified by removing statistically insignificant terms [22] such as the quadratic terms leading to a two-factor-interaction (2FI) model (Equation (7)):

(7) $$Y=\beta_{0}+\sum \beta_{i}x_{i}+\sum \beta_{ij}x_{i}x_{j},$$

The Design expert (Version 8.0.2, Stat-Ease Inc., Minneapolis, MN 55413, USA) was used to statistically evaluate the model’s suitability, execute the analysis of variance (ANOVA) test, and produce the response surface graphs.

The linear correlations between the severity factors (SF), the PHWE variables, and the TPA responses were further investigated using a Pearson moment correlation (Table S1). The Pearson correlation tests were performed using Statgraphics Centurion XVIII, version 18, software (2017, StatPoint, Inc., Warrenton, VA, USA).

The severity factor, commonly referred to as log (*R_0_*), measures the intensity of the PHWE crude agar recovery process and is calculated using the following equation (Equation (8)):

(8) $$\log(R_{0})=log\left\{t\times e^{\left[\frac{\left(T-100\right)}{14.75}\right]}\right\},$$

where *T* is the temperature (°C) and *t* is the time (min). The severity factor (Table 1) ranged from 1.06 (run 24: T = 80 °C and time = 45 min) and 3.06 (run 22: T = 130 °C and time = 150 min).

Design Expert 8 was used to find the multi-response optimum conditions using the desirability test, which consists of reducing multicriteria problems to a single desirable score. The approach consists of measuring the partial desirability function (*di*) for each response which runs from zero, the least desirable response, to one, the optimal response. The global multiresponse desirability (*D*), which is defined as the average weighted geometric of *n* individual desirability functions, was then obtained from Equation (9) [23].

(9) $$D={\left[\prod_{i=1}^{n}d_{i}{}^{Pi}\right]}^{\frac{1}{n}},$$

where *Pi* is the weight of the response, normalized so that$\;\overset{n}{\underset{i}{\sum }}Pi=1$. The ideal optimization considers the relative importance of each response while selecting the most appropriate form of the partial desirability function (Table S2).

## 3. Results and Discussion

The most suitable data analysis approach was determined by the sequential sum of squares, the lack of fit, and the summary statistic tests. The 2-factor interaction (2FI) model was strongly suggested, while the quadratic and higher models were aliased. The statistical significance of the 2FI model was associated with an insignificant lack of fit at a 95% confidence level for all textural responses as demonstrated by the ANOVA tests (Table 2).

The dependency of the textural responses of the recovered gels on the PHWE operating factors was, therefore, evaluated using the 2FI model. The extraction was carried out over a wide range of severity factors (SF) by synergistically interacting various temperatures and extraction times. At an SF of 2.5 and higher (Table 1), however, the structure of the recovered agar did not withstand the double compression test (Figure 2). Accordingly, the response surface modeling took into consideration the impact of the excluded data resulting from the limit of data collection. In this work, the level for each PHWE operating factor was assigned based on a previous study [15] that evaluated their impact on the agar recovery process. The recovery process of the *Gelidium sesquipedale* industrial waste stream via PHWE resulted in residual crude agar yield ranging from 10 ± 2 to 17 ± 2%.

This yield was substantially high as compared to those reported by authors such as Trigueros et al. [24] who found a 5% agar yield from a semi-continuous pressurized hot water extraction of the algae by-products. This higher yield could be explained by the lowering of the agar hydrolysis process due to the use of moderate process temperatures (80 ≤ T °C ≤ 130 °C) and shorter extraction times (45 ≤ t ≤ 150 min).

From a physical point of view, the strength of the agar gel was found to significantly vary with temperature, pressure, time, and algae content. The gel strength of the PHWE recovered agar fluctuated between a minimum of 25 g/cm^2^ to a maximum of 350 g/cm^2^ value for a 1.5% (*w:v*) agar solution. It agreed with the findings of Martnez-Sanz et al. [25] who found values of 245 10 g/cm^2^ and 275 10 g/cm^2^ for unpurified agar derived from non-pretreated algae for agar extracted using hot water and a combination of heating and sonication, respectively. The high strengths reported for commercial agar gel (>700 g/cm^2^ in a 1.5 wt.% solution) [26] are commonly achieved following preliminary pretreatment of the seaweed and further purification of the agar. Authors such as Diop et al. [15] have shown that the PHWE parameter accounted for 84.27 percent of the variation in gel strength, according to the mathematical model of the response surface. The depolymerization caused by water’s ability to hydrolyze itself at high temperatures and pressures may be a factor in the gel’s molecular structure’s stability. As for the texture, the variability of gel strength has been in high demand in a wide range of applications, including in food processing.

**Figure 3:**
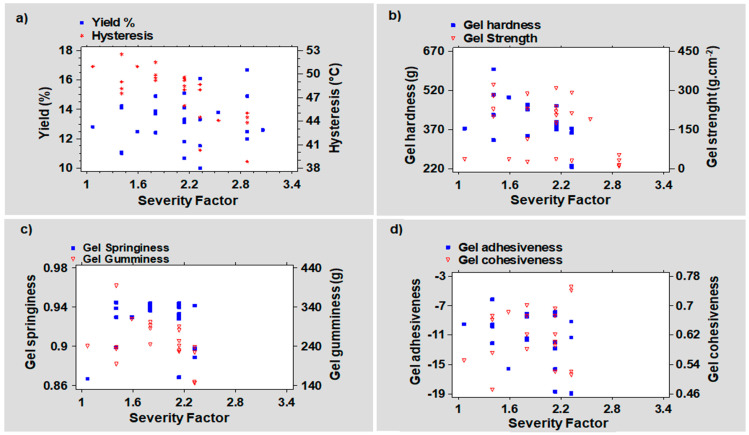
Correlations between the severity factor of the pressurized hot water extraction treatment and (**a**) the agar yield (%) and hysteresis (°C), (**b**) the agar gel hardness (g) and gel strength (g.cm^−2^), (**c**) the agar gel hardness (g) and gel strength (g.cm^−2^), and (**d**) the gel adhesiveness and cohesiveness.

On the other hand, the thermal hysteresis signifying the gap between the melting and the gelling temperatures was found to oscillate between 39 ± 1 and 52 ± 0.5 °C. When exposed to severe treatments, this wide variation may be related to a positional change of the hydrogen bonds in the polymer chains [27]. Figure 3 correlates the physical (Figure 3a,b) and textural properties (Figure 3c,d) of the agar gel in the function of the severity factor (SF) of the PHWE process.

**Figure 4:**
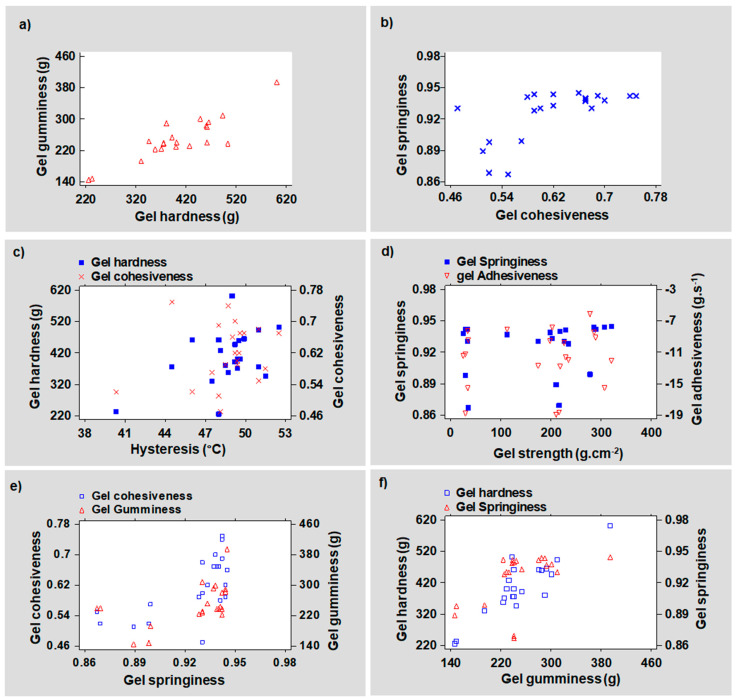
Correlations between tested physical parameters of the crude agar gel recovered from the algae industry waste stream via pressurized hot water extraction (PHWE). (**a**) Linear relationship between the agar gel gumminess and hardness. (**b**) Linear correlation between the agar gel springiness and cohesiveness. (**c**) Correlations between the hysteresis (°C) of the agar gel and its hardness (g) and cohesiveness. (**d**) Linear relationship between the agar gel strength (g.cm^−2^) and its springiness and cohesiveness (g.s^−1^). (**e**) Correlation between the recovered agar gel springiness and the gel cohesiveness and gel gumminess (g). (**f**) Relationship between the agar gel springiness, the gel cohesiveness (g.s^−1^) and the gel gumminess (g).

With a coefficient (r) of −0.50 and *p*-value (*p*) of 0.0185, the Pearson moment demonstrated a strong negative and significant linear relationship between the severity factor and the gel hysteresis (Table S1). It corroborates the negative influence of intensive PHWE operating conditions on both the strength and the thermal hysteresis of the gel. Figure 4 displays the linear correlation between the physical properties of the gel and its TPA parameters. A moderate and significant positive relationship was found between the thermal hysteresis of the agar and its gel texture, principally, its hardness (r = 0.47, *p =* 0.0333) (Figure 4c).

**Figure 5:**
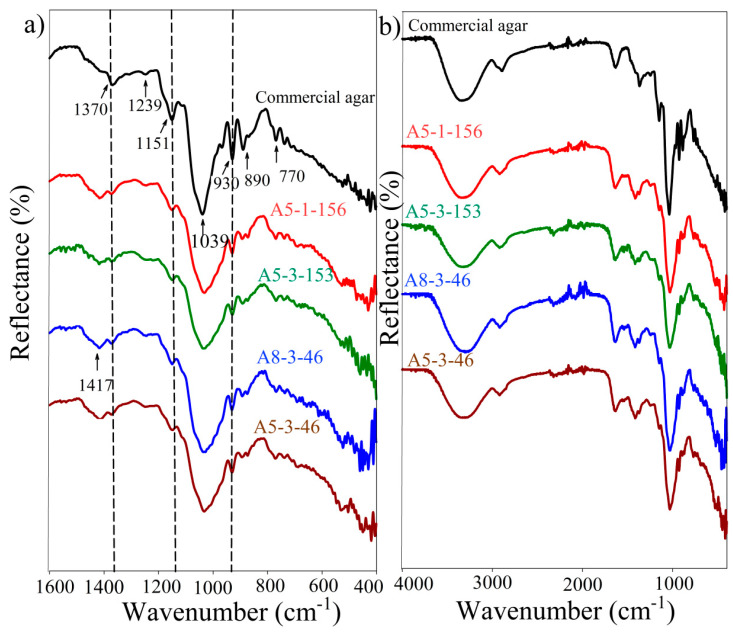
FT-IR spectra of the commercial and pressurized hot water extracted agar: A5-1-156 (temperature (A) = 105C, pressure (B) = 1 bar, recovery time (C) = 150 min, and algae-to-water ratio (D) = 6.5%)—A5-1-153 (A = 105 C, B= 1 bar, C = 150 min, and D = 5% (*w:v*))—A8-3-46 (A = 80C, B = 37.5 bar, C = 40 min, and D = 6.5% (*w:v*) –A5-3-46 (A = 105C, B = 37.5 bar, D = 40 min, and C = 6.5% (*w:v*)). (**a**) wavenumber between 1600–400 cm^−1^ and (**b**) wavenumber between 4000–400 cm^−1^.

Figure 5 depicts the FT-IR spectra of commercial and crude agar recovered via the PHWE of the algae industry waste. A broad band was observed at around 3300 cm^−1^ in all spectra and was attributed to the combined stretching vibrations of NH and OH groups [28] (Figure 5b). It also confirmed a substantial influence of the water content in the samples. The asymmetric (CH) stretching modes could be attributed to the peak at 2920 cm^−1^, however, the band at 1634 cm^−1^ could be assigned to the moisture water bending (HOH) vibrations. The bending (NH_2_) and stretching (C=O) modes could also interfere in this region [29]. The typical peaks of agar are principally found in the wavenumber range between 400 cm^−1^ to 1600 cm^−1^ (Figure 5a). The wagging (CH) vibrational modes for both sulfated and unsulfated agarose [30] have been attributed to the band at 1370 cm^−1^ [31]. On the other hand, the weak band at 1239 cm^−1^ could be attributable to the interrelated influence of peaks at around 1250 cm^−1^ assigned to pyranose ring (CH_2_) bending vibrations in agarose and an absorption appearing at about 1220 cm^−1^ [30] which could be caused by ester-sulfate groups. The absorption between 1409 and 1417 cm^−1^ could be attributed to the (CH_2_) modes, while the peak at 1039 cm^−1^ was associated with the CO and COC vibrations of the 3,6-anhydrogalactose bridge as well as the glycosidic linkage [32]. Peaks emerging between 1000 and 1100 cm^−1^ were assumed to be typical of the CO and OCH vibrations of polysaccharides such as agarose. Agarose characteristic bands were perceived at 930, 872, and 770 cm^−1^, attributed to the 3,6-anhydro-galactose skeletal bending modes [33]. The peak at 890 cm^−1^, on the other hand, was attributed to both the CC and CO vibrations in the 1,3-linked -galactopyranosyl units.

### 3.1. Impact of the PHWE on Gel Hardness

The hardness of the recovered crude gel was shown to be significantly dependent on the variations of PHWE temperature (*p =* 0.0075) and time (*p =* 0.0013) according to the ANOVA results. The two-way interaction between temperature and time (*p =* 0.0042) and the two-way interaction between temperature and the algae-to-water ratio (*p =* 0.0009) were both statistically significant. The interrelation between the pressure and the extraction time was also found to be significant (*p =* 0.0114). Even though all the variables were tested at a 5% tolerance level, the greatest impact on the gel stiffness was found to be related to the interaction between temperature and algae concentration. Nonetheless, 82% of the observed variation in the recovered agar gel hardness was roughly explained by the proposed experimental model as indicated by the R-squared (R^2^) value. The adjusted R-squared (adj-R^2^) was equal to 72%, therefore, a coded regression equation (Equation (10)) was generated from the 2FI model in Table 3.

In response to the PHWE factor variations, the relationship between the predicted values and the actual experimental values of the gel texture parameters was determined (Figure S1). Crude agar gel’s hardness ranged from 601 g to 225 g (Table 1). Lower agar gel hardness values were reported by Rebello et al. [34] for the red algae *Gracilaria*. These authors reported values that oscillated between 15 and 270 g for an untreated and a 10% alkaline treated *Gacilaria,* respectively. The maximum hardness of the PHWE recovered crude agar obtained in the present study was 2.23 times higher than the maximum hardness of the alkaline-treated *Gracilaria* agar. It is, however, necessary to mention structure variabilities between Rhodophyta *Gracilaria* and *Gelidium*, despite the scarcity of available textural data for the latter.

**Figure 6:**
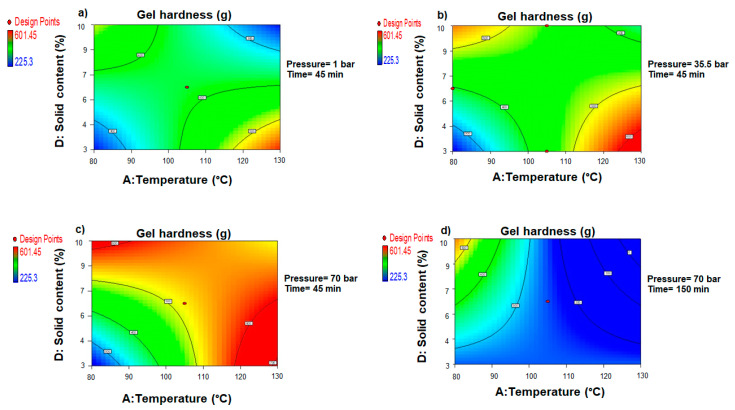
Response surface contour plots showing the combined influences of the pressurized hot water extraction (PHWE) temperature (A) and solid content (D) on the hardness of the recovered agar gel at fixed pressure (B) and the operating time (D). (**a**) B = 1 bar and C = 45 min. (**b**) B = 35.5 bar and C = 45 min. (**c**) B = 70 bar and C = 45 min. (**d**) B = 70 bar and C = 45 min.

Response surface contour plots (Figure 6) were used to show the combined effects of temperature, time, pressure, and algae-to-water ratio on the hardness of the agar gels recovered from the *Gelidium sesquipedale* waste stream. The rapid recovery process (factor C = 45 min) (Figure 6a) was tested at atmospheric pressure (B = 1 bar).

The results revealed that in these conditions, elevated temperatures and low algae-to-water ratios were required to recover harder agar gels. Increasing the water pressure to 35.5 bar without prolonging the recovery time, on the other hand, resulted in a distinct configuration (Figure 6b). This new pattern, which required the combination of a high algae concentration and a very low processing temperature, especially favored the production of a harder gel texture. It was deduced from the contour plots that, while not statistically significant, the pressure influence on gel hardness was not completely negligible. Increasing the internal pressure to 70 bar with a short operating time corroborated this assumption and resulted in a visible expansion of the favorable experimental region (Figure 6c). Within these experimental settings, recovery of agar with harder gels was mainly favored, depending on the extraction temperature and the algae concentration.

It is also worth noting that pressure had a particularly positive influence on the stiffness of the gel when associated with short processing times. Extending the time to 150 min while maintaining high-pressure conditions (Figure 6d) resulted in a completely reversed trend, with gel hardness substantially decreasing within the experimental zone.

The severity factor of the PHWE process had a moderately negative linear relationship with the resultant gel hardness, according to the Pearson-moment correlation. Despite being statistically significant (*p =* 0.0333), this relationship had a negative Pearson coefficient (r = −0.46). This negative relationship, on the other hand, was consistent with the above-observed phenomenon that linked the incapacity of the recovered agar gels to withstand a double compression to SF above 2.5 (Table 1).

At subcritical conditions, the formation of H_3_O^+^ ions due to water’s self-ionization tends to acidify the medium, conferring to water an autocatalytic power [35]. As the operating time is extended, the rate of partial hydrolysis undergone by agar molecules increases as a result of phase transition, causing structural changes in the gel. Applying severe recovery conditions was found to lower the values of some textural parameters such as hardness (Figure 3b). On the other hand, the linear relationship between the recovered agar gel hardness and the gel strength (r = 042, *p* > 0.05) was not statistically significant. However, a moderately positive and statistically significant relationship between gel hysteresis and gel hardness was found using the Pearson correlation (r = 0.47, *p =* 0.0333) (Figure 4c). Furthermore, operating at low algae concentration and atmospheric pressure clearly reveals that two distinct temperature and extraction time combinations were associated with the extraction of a crude agar with a harder gel texture. It was crucial to run the process at a low temperature and long extraction period, or an elevated temperature with a shorter processing time, in relation to the reduced severity factor. At temperatures above 110 °C, however, pressurizing the water in contact with lower content of algae tended to restrict the optimum region to reaction times ≤75 min. In contrast, when the algae to water ratios were increased from 3 to 10% (*w:v*), the impact of the recovery time on the gel hardness appeared to be less decisive, even though keeping the temperature below 90 °C was crucial. The molecular degradation that occurs in severe PHWE settings may have an impact on the helical structure cohesive strength in the crude agar gel [36].

### 3.2. Impact of the PHWE on the Gel Cohesiveness

The cohesiveness of material determines how homogeneous it is and how much it can be deformed before rupturing. The ratio of total energy at the first compression to the total energy at the second compression was translated mathematically in Equation (4).

The 2FI model’s suitability was strengthened by its strong statistical significance (*p =* 0.0001) at a 5% tolerance level with an insignificant lack of fit. Based on the Box-Behnken design, the most significant impacts on the cohesiveness of recovered crude agar gel were induced by the pressure (*p =* 0.0076) and the algae to water ratio (*p =* 0.0028), according to the ANOVA test. While the singular effects of temperature and recovery time were statistically insignificant, the interactions of the former parameter with its PHWE factor counterparts were crucial to the gel’s textural properties. As a result, there was a significant two-way interaction between the temperature and the pressure (*p =* 0.0263), the temperature and the extraction time (*p =* 0.0153), and, more particularly, the temperature and the ratio of algae-to-water (*p =* 0.0048). Similarly, the interaction between the pressure and extraction time (*p =* 0.0003), as well as the recovery time and the algae concentration (*p =* 0.0006), were crucial in generating a more cohesive gel.

The cohesiveness values of the PHWE recovered agar gels ranged from 0.47 to 0.75 (Table 1). While there are few studies on *Gelidium* agar, Yarnpakdee et al. [37] reported that the red algae *Gracilaria tenuistipitata* showed similar gel cohesiveness. The authors reported values of 0.45 ± 0.008 and 0.75 ± 0.000 for commercial and native untreated agar, respectively.

**Figure 7:**
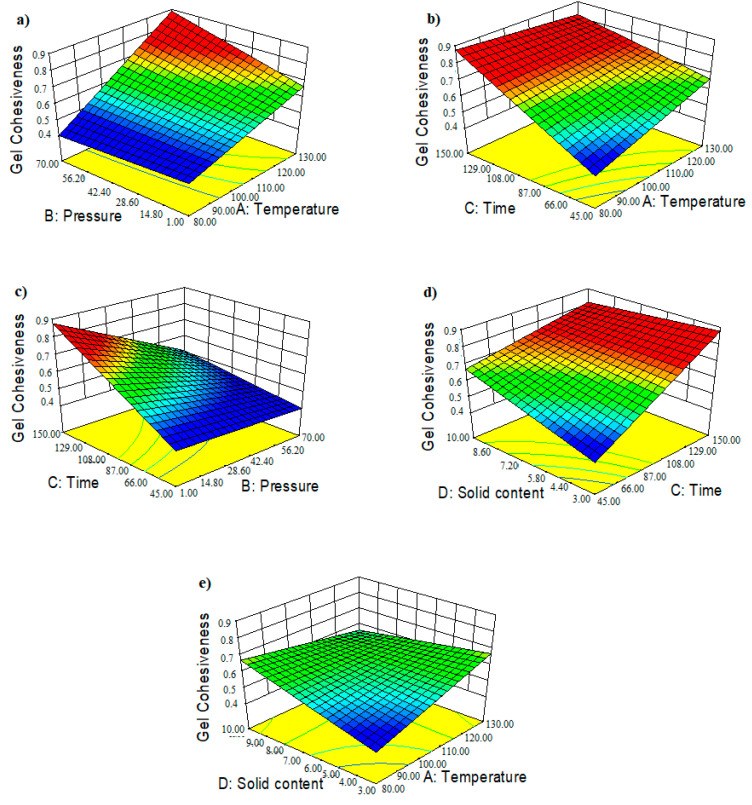
Three-dimensional (3D) response surface contour plots showing the combined effects of the (**a**) the pressurized hot water extraction (PHWE) temperature and pressure, (**b**) the recovery time and temperature, (**c**) the recovery time and pressure, (**d**) the solid content and recovery time and (**e**) the solid content and temperature on the recovered agar gel cohesiveness.

The predicted gel cohesiveness values from the fitted mathematical model were compared to the actual TPA experimental data (Figure S1b). The fitted polynomial 2FI model’s coded regression equation (Equation (11)) was shown in Table 3 and the model explained 91% (R^2^ = 91%) of the observed variability in gel cohesiveness, with an adjusted R^2^ (adj-R^2^) value of 83%. The 3D response surface (Figure 7a–e) and contour plots (Figure S2) depicting the combined effects of the PHWE parameters on the cohesiveness of the recovered crude agar gels were also obtained.

Therefore, depending on the pressure, recovering crude agars that form a high cohesive gel required elevated operating temperatures associated with short recovery times. Consequently, the probability of achieving a satisfactory cohesiveness at atmospheric pressure (B ≈ 1 bar) during a fast recovery was extremely low. When high temperatures (A > 120 °C) were combined with an algae concentration (D) of less than 8% (*w:v*), a subsequent increase in pressure to 35.5 bar seemed to improve the gel homogeneity. Higher-pressure processing (B = 70 bar) at temperatures above 110 °C (Figure 7a), on the other hand, considerably enhanced the gel cohesiveness of the recovered agar within 45 min, regardless of the algae concentration (Figure S2c).

Alternatively, lengthening the PHWE treatment time from 45 to 150 min at atmospheric pressure could notably increase the gel homogeneity (Figure 7b). High algae to liquid ratios combined with a temperature above 92 °C, nonetheless, tended to inhibit this alternative. Fujimoto et al. [36] found that, in addition to the cohesive force between particles, gel cohesiveness was substantially related to the agarose molecular weight. The molecular weight of agarose was reported to be lower than that of the agaropectin fraction in the same agar molecule [38]. As a result, hydrolysis occurring at intense PHWE treatments tends to primarily alter the molecular weight of the agarose fraction.

This mechanism reduces molecular cohesion by weakening hydrogen bonding in the agar gel structure in connection to the double-helical formation [39]. Pressurization, on the other hand, was proven to considerably limit the possibilities of creating conditions that improved gel cohesiveness at extended recovery time. Under this assumption, producing a suitable cohesive gel became nearly impossible when a pressure of 70 bar was combined with a 150 min extraction time, regardless of the temperature or algae concentration.

In a similar pattern, the production of highly cohesive gels with a 3% (*w:v*) algae-to-water ratio and a low process temperature (80 °C) requires a PHWE time of more than 108 min and a pressure of less than 28 bar. The application of a process temperature of around 130 °C, however, appears to significantly broaden the favorable extraction region, allowing for high cohesiveness while maintaining a low solid concentration.

In contrast to a lower algae concentration, generating a high homogeneous gel at a high solid-to-liquid ratio (10% *w:v*) required low temperatures around 80 °C, long recovery times, and pressure below 21 bar. In those conditions, an increased temperature (A = 130 °C) created an inverse trend, necessitating elevated pressure (B > 50 bar) and an extraction time of less than 65 min (Figure S2f). The linear relationship between gel hardness and cohesiveness was significant (*p* > 5%), despite the correlation being found to be moderately positive (r = 0.42) (Table S1). The combination of these two texture responses (Equation (5)) may reveal substantial information about the gumminess of the recovered agar gel. In contrast, an insignificant positive weak Pearson correlation (r = 0.23, *p* > 5%) between the gel’s thermal hysteresis and its cohesiveness can be linked to Figure 4c.

### 3.3. Impact of the PHWE on the Gel Gumminess

Gumminess is a property of semisolid materials that have a low degree of hardness and a high degree of cohesiveness [40]. The two-factor interaction (2FI) model was strongly significant with a low *p*-value (*p =* 0.0004) and an insignificant lack of fit (*p =* 0.0639). The model suitably evaluated the impact of PHWE factors on the recovered crude agar gel gumminess due to the aliasing of higher models. The ANOVA test showed that the reaction time (*p =* 0.0005) was the only singular factor with enough evidence of an impact on gumminess at a 5% tolerance level. However, as shown in Table 2, the two-way interactions between the different PHWE operating factors had a considerable impact on the gumminess of the crude agar gels. As a result, the interrelations between temperature and the recovery time (*p =* 0.0032), pressure and extraction time (*p =* 0.0002), and temperature and algae-to-water ratio (*p =* 0.0003) were statistically significant. The obtained R^2^ value suggested that the model can explain 82.5% of the observed variability in gel gumminess values, with an adj-R^2^ of 73.2%. Table 3 shows the coded regression equation (Equation (12)) based on the polynomial 2FI approach.

The mathematically predicted values of residual agar gel gumminess were plotted against the actual experimental values (Figure S1c). The experimental values for gumminess ranged from 147 g to 395 g (Table 1).

**Figure 8:**
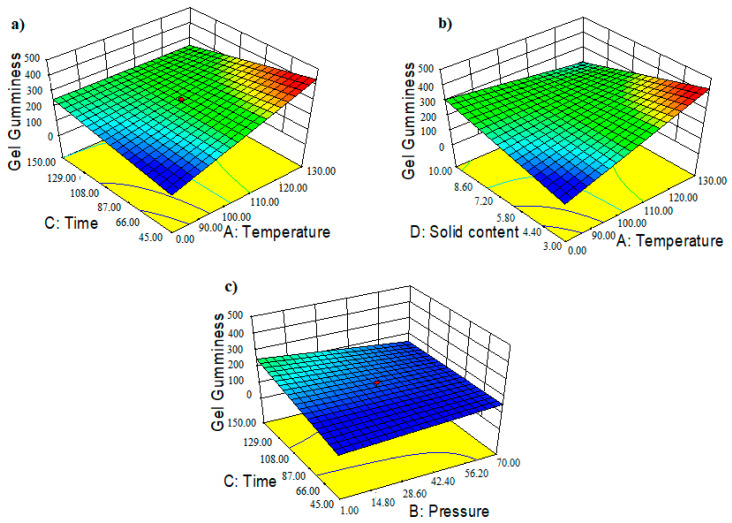
Three-dimensional (3D) response surface contour plots showing the combined effects of (**a**) the operating time and temperature of the pressurized hot water extraction (PHWE), (**b**) the solid content and temperature and (**c**) time and pressure on the recovered agar gel.

The evolution of gumminess as a function of the two-way interaction between the PHWE variables was depicted in the 3D response surface (Figure 8a–c) and the response surface contour plots (Figure S3) which show the variation of gel gumminess as a function of temperature and time. The results showed that increasing the temperature resulted in the recovery of crude agar grades with a high level of gumminess when the algae-to-water ratios were low (D = 3% *w:v*). Technically, a lower solid-to-liquid ratio in the extractor tended to promote a better algae dispersion in the pressurized hot water. A greater solid dispersion improves mass transfer by increasing accessibility and speeding up the diffusion rate of the free solvent [41]. The chosen model revealed that gel gumminess was markedly enhanced when high temperatures (A ≈ 130 °C) were combined with reaction times shorter than 100 min. It was clearly different from the very low response observed at low temperature (A ≈ 80 °C) at the same reduced algae-to-water ratio, regardless of the extraction time or pressure applied. Once the algae concentration was increased to 10% (*w:v*), lowering the recovery temperature and pressure while using long recovery times improved the material gumminess. In other words, the lower the gumminess, the higher the process temperature was at this high solid concentration, especially when extended recovery times were involved. The Pearson correlation demonstrated a strong positive linear relationship between crude agar gel hardness and gumminess (r = 0.88, *p =* 0.0001) (Figure 4a). However, a moderately significant positive correlation was discovered between gumminess and gel cohesiveness (r = 0.48, *p =* 0.0275) (Table S1). The severity factor, on the other hand, had a weak negative relationship (r = −0.37) with the obtained gel gumminess, although was statistically insignificant (*p* > 5%). Agar gel gumminess (r = 0.43, *p* > 5%) and thermal hysteresis showed a moderately positive correlation but were statistically insignificant When the PHWE recovery time was extended, however, the synergistic interaction between internal pressure and algae concentration showed a crucial impact on the gel gumminess. As a result, a proportionate relationship seemed to indicate that high-pressure processing should be combined with a high solid-to-liquid ratio and a lower temperature to maintain a suitable gumminess of the gel. High-pressure processing has also been linked to an increase in the gumminess of a semi-solid material by Yusof et al. [42]. Furthermore, the gumminess values of pressure-induced gels were found to be lower than those of the heat-induced gel according to Okamoto et al. [43].

### 3.4. Impact of the PHWE on the Gel Adhesiveness

The negative strength [44] required to overcome the attractive force and pull the compressive probe away from the gel surface is referred to as adhesiveness [45]. The 2FI was the suggested statistical approach to analyze the impact of the process factors on the repelling force of the gel. The model’s suitability was proven by a high statistical significance (*p* < 0.0001) and an insignificant lack of fit (*p =* 0.1087).

The ANOVA test (Table 2) showed that all individual PHWE factors, with the exception of pressure, were statistically significant in inducing an adhesiveness response. The variation in the recovery time (*p* < 0.0001) and the ratio of algae-to-water (*p* < 0.0001) appeared to have the most significant impacts on the change in adhesiveness of the crude gel. The two-way interaction between the pressure and the extraction time (*p =* 0.0009) appeared to have a significant effect on the adhesiveness of the residual agar gel, similar to the interaction between time and algae concentration (*p =* 0.0253). The chosen 2FI model (adj-R^2^ = 86%) was responsible for 93% (R^2^) of the repulsive energy variation observed and the magnitude of the negative area ranged from −19 to −6 g. s^−1^ (Table 1). As a result, the predicted force values were based on the fitted regression equation (Equation (13)) (Table 3) against the experimental adhesiveness data (Figure S1d).

When operating at a low temperature of 80 °C and a low algae-to-water ratio of 3% (*w:v*), an interesting response pattern was observed. This pattern demonstrated the prevalence of a gel with low adhesiveness (Figure S4a) when the process was carried out under these conditions. With the exception of conditions involving the combination of long recovery time (C > 129 min) with a pressure higher than 55 bar, this pattern was preeminent across the entire experimental range. Results showed that raising the temperature to around 130 °C resulted in an inversed pattern, restricting the recovery of agar with reduced adhesiveness to two distinctive combinations of time and pressure. As a result, lower adhesiveness might be obtained either at high temperatures by using high pressure and a short extraction time (less than 60 min) or by using long extraction times at low pressures (under 7 bar). It is worth mentioning, however, that the aforementioned trends were associated with an algae concentration not exceeding 3% (*w:v*). Sousa et al. [45] have linked an improvement in the agar extraction process to an increase in solvation and diffusion capacities of the solvent. Increasing the solid-to-liquid ratio to 10% (*w:v*) appears to significantly accelerate the production of agar gel with higher adhesiveness, as demonstrated by a larger negative area, regardless of the operating temperatures (Figure S4c,d).

The effect of the pressure was negligible on the gel adhesiveness according to the ANOVA test. However, it has been shown that the impact of pressure’s interactions with its other PHWE parameter counterparts, particularly the extraction time, was substantial. Therefore, combining atmospheric pressure, long extraction time, and low algae concentration was required to reach an optimal recovery zone. Therefore, subsequent pressurization of the hot water tended to shift the positive experimental pattern in the opposite direction. Consequently, a region requiring a combination of short extraction times and higher algae-to-water ratios was created. On the other hand, the ability to recover reduced adhesiveness was inversely proportional to the amplitude of the operating temperature. As illustrated in Figure 4d, the adhesiveness appeared to be related to the gel strength. A decrease in agar gel strength is frequently observed at high severity factors [46]. Nonetheless, the Pearson moment correlation seemed to indicate that the linear relationship between the severity factor and the gel adhesiveness was not statistically significant at a 5% tolerance level (*p =* 0.566). Conversely, as shown in Figure 3d, the Pearson coefficient (r = −0.42) indicated a moderate negative impact of the SF on adhesiveness.

### 3.5. Impact of the PHWE on Gel Springiness

The height at which the agar gel recovers after the first compression and before the second compression during a TPA test is known as springiness and is associated with the material’s elasticity.

The two-factor interaction model was the most significant (*p =* 0.0033) among the tested analytical approaches at a 95% confidence level. The model’s pertinence was validated by the statistically insignificant lack of fit (*p =* 0.1423) when compared to advanced models including the aliased quadratic approach. According to the ANOVA results (Table 2), both the applied pressure (*p =* 0.0081) and the algae-to-water percent ratio (*p =* 0.0021) had significant influences on the gel springiness. The two-way interactions between process temperature and recovery time (*p =* 0.0036), temperature and algae-to-water ratio (*p =* 0.0088), and applied pressure and algae concentration (*p =* 0.0491) were all statistically significant.

The 2FI model explained 75.6% (R^2^) of the variability of the gel springiness (adj-R^2^ = 62%). As a result, the experimental values obtained from the PHWE recovery process ranged from 0.86 to 0.95. Whereas data on *Gelidium* agar is limited, Yarnpakdee et al. [37] found that commercial agar has a springiness value of 0.81, while native agar from *Gracilaria* had a value of 0.92. The fitted regression equation (Equation (14)) developed based on the polynomial multifactorial model was given in Table 3.

**Figure 9:**
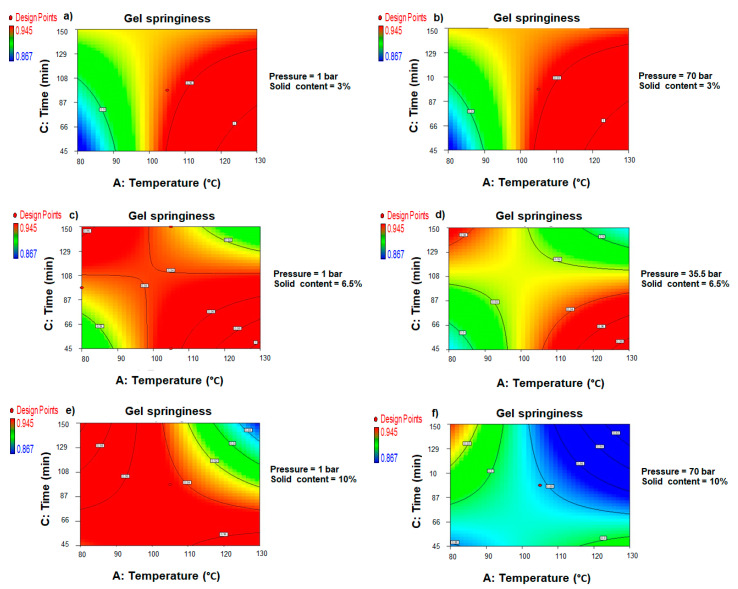
Response surface contour plots showing the combined influences of the pressurized hot water extraction (PHWE) temperature (A) and operating time (C), on the springiness of the recovered agar gels at fixed pressure (B) and solid content (D). (**a**) B = 1bar and D = 3% (*w:v*). (**b**) B = 70 bar and D = 3% (*w:v*), (**c**) B = 1 bar and D = 6.5% (*w:v*). (**d**) B = 35.5 bar and D = 6.5% (*w:v*), (**e**) B = 1 bar and D = 10% (*w:v*) and (**f**) B = 70 bar and D = 10% (*w:v*).

The agar gel springiness values were mathematically predicted using a multivariate polynomial equation (Equation (14)) and plotted against the experimental data (Figure S1e). The most important individual factor impacting the elasticity of the produced gel was found to be pressure. Figure S5 shows the 3D response surface plots illustrating the interrelated effects of the PHWE parameters on the springiness response of the recovered agar gel. The combined effects of the PHWE parameters on gel springiness are represented in the response surface contour plots (Figure 9). 

When PHWE was operated at a low ratio of algae-to-water (D = 3% *w:v*), however, injecting pressure in the extractor had no noticeable effect on the variation of the gel elasticity. At this operating stage, higher elastic gels were formed at temperatures above the water’s boiling point (100 °C), regardless of the extraction duration or the pressure used (Figure 9a,b). At low pressure (B = 1 bar), the effect of the algae concentration on the ability of the agar gels to recover a suitable height after the first compression was more visible. At this pressure level (Figure 9c), raising the algae concentration to 6.5% (*w:v*) caused the optimum experimental region to show a symmetrical diagonal expansion. The combination of high temperatures (A > 95 °C) and processing times less than 120 min, as well as low operating temperatures (A < 95 °C) and long extraction times (C > 90 min), formed this region. With the pressure rise of 35.5 bar, the interconnectivity between the two regions (Figure 9d) was reduced within a persistent optimum pattern. With the increase in the algae concentration to 10% (*w:v*), the unfavorable experimental zone appeared to be circumscribed to an exclusive area requiring temperatures over 105 °C and extraction times longer than 95 min (Figure 9e). Any condition excluded from this specific area supported the production of agar with improved springiness when a pressure of 1 bar was used in the agar recovery process. However, from higher-pressure processing (B = 70 bar), it was demonstrated that raising the algae-to-water ratio to 10% (*w:v*) decreased the springiness of the agar gel (Figure 9f). Temperatures below 85 °C in combination with a long extraction time (C > 130 min) were the only conditions allowing acceptable elasticity of the recovered gel.

When lengthy recovery periods were considered, the higher the applied pressure, the lower the required temperature, and the higher the solid concentration required to impart enhanced gel springiness. In these conditions, the flexibility of the recovered crude agar gel appeared to be reduced while working at high pressure.

Furthermore, RSM linked the optimum elastic potential of the recovered agar to higher temperatures during short process times. Conversely, in most pressurized conditions, the springiness values decreased when a low temperature (80 °C) was combined with a shortened recovery time. As a result, the springiness of the recovered agar gel was favored when the PHWE process time was extended to 150 min at atmospheric pressure. However, it was only achievable when lower temperatures (A < 100 °C) and a solid concentration greater than 4% (*w:v*) were involved. Following a similar trend, Suzuki et al. [39] demonstrated a linear correlation between the molecular breakdown of agar and its gel structure. Because the material’s springiness is inversely proportional to its hardness, the more rigid the agar gel was, the less elastic it was [45]. As expected, an intensification of operating conditions negatively impacted the gel springiness due to biopolymer depolymerization. It also could weaken the gel structure due to a lower agar molecular weight. On the other hand, the Pearson correlation only confirmed a non-significant but weak positive relationship between the severity factor and gel springiness (r = 0.13, *p* > 5%) (Figure 3c). The linear Pearson correlations found between the residual agar gel’s springiness and, respectively, its cohesiveness (r = 0.65, *p =* 0.0014) (Figure 4b) and gumminess (r = 0.52, *p =* 0.0161) (Figure 4f) were statistically significant and strongly positive.

### 3.6. Desirability of the PHWE Process

**Figure 10:**
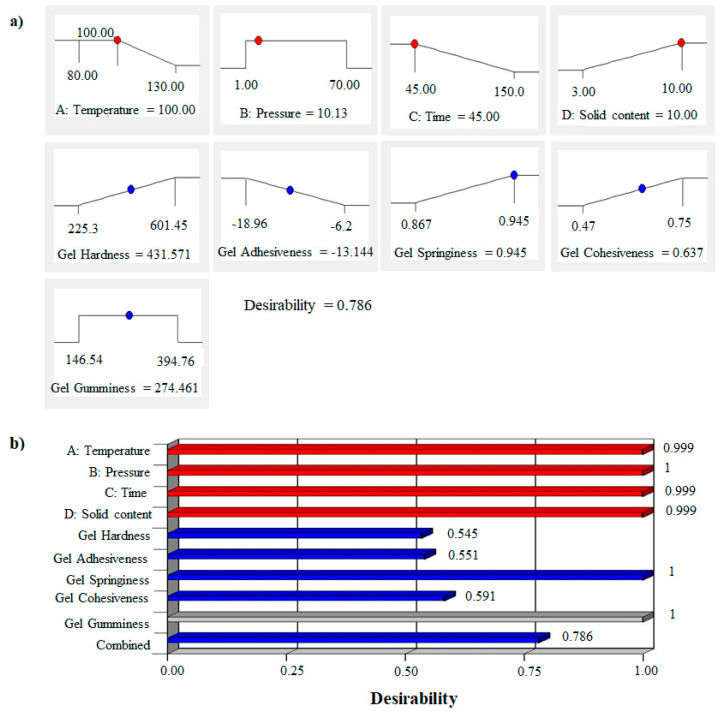
(**a**) Ramp function graph of pressurized hot water extraction process parameters targeting a minimization of the temperature and recovery time while maximizing the algae-to-water percent ratio. (**b**) Bar graph of desirability for combined optimization of the PHWE process.

The desirability test was carried out to determine an optimal geometric arrangement for all four key PHWE parameters considered in this study. An optimal combination of the PHWE operating factors was provided based on the textural responses of the recovered crude agar. Desirability near 1 [23] was considered the most effective value close to the target set for each parameter and response (Table S2). The aim was to adjust the textural properties of the gel while increasing the algae concentration in the PHWE reactor throughout the recovery process and reduce the severity of the treatment by lowering the temperature and the operating time. The ramp function and desirability bar graphs represented in Figure 10a,b display the elementary and combined desirability as well as the optimum recovery conditions.

A temperature of 100 °C, combined with a pressure of 10.13 bar, a short recovery time of 45 min, and an algae-to-water ratio of 10% (*w:v*) were found to be optimal PHWE parameters values. The evaluation of each optimized textural response under these conditions was reported in Figure 10a. Furthermore, a global optimization was carried out using a mathematical tool to integrate each of these individual responses. The bar graphs (Figure 10b) show the proficiency of each response by comparing their proximity to 1. Therefore, the combined multiresponse desirability of 0.786 was found to be attractive.

## 4. Conclusions

The impact of pressurized hot water extraction (PHWE) parameters on the cascade biorefining process of discarded *Gelidium sesquipedale* industrial waste stream has been studied. Response surface analysis has shown that negative impacts on the texture profile of the recovered residual agar gel were induced by severe PHWE treatments that caused partial molecular hydrolysis. The PHWE process permitted recovery of between 10 ± 2 to 17 ± 2% of residual crude agar from the waste stream. Pearson correlation confirmed the negative relationships between the severity factor and all texture parameters. The adjustability of the generated textures will expand the spectrum applications of residual agar. The best PHWE parameter values were discovered to be a temperature of 100 °C combined with a pressure of 10.13 bar, a short recovery time of 45 min, and an algae-to-water ratio of 10% (*w:v*). The process can produce agar with a hardness value of 431.57 g, an adhesiveness of −13.14, a cohesiveness of 0.63, a springiness of 0.94, and a gumminess value of 274.46 g based on the Assigned process desirability. In the framework of circular economy and industrial symbiosis, the selectivity of PHWE is an advantageous strategy for the valorization of agri-industry waste.

## Figures and Tables

**Table 1:** The experimental pattern of the Box-Behnken design reporting the different process factors and response values of the gel hardness (g), gel adhesiveness (g.s^−1^), gel cohesiveness, gel springiness (mm), and gel gumminess (g) of the residual agar obtained from the pressurized hot water extraction (PHWE) process of the never-dried algae industry waste-stream.

	Factor A	Factor B	Factor C	Factor D			Response 1	Response 2	Response 3	Response 4	Response 5
Run	Temperature (°C)	Pressure (bar)	Time (min)	Solid Content (*w:v*%)	Severity Factor	Yield (%)	Hardness (g)	Adhesiveness (g.s^−1^)	Cohesiveness	Springiness (mm)	Gumminess (g)
1	105 (0)	35.5 (0)	45 (−1)	3 (−1)	1.80 ^c^	14.9	460.2	−8.55	0.62	0.944	284.65
2	80 (−1)	70 (1)	97.5 (0)	6.5 (0)	1.40 ^c^	14.1	427.01	−9.83	0.47	0.93	232.58
3	105 (0)	35.5 (0)	45 (−1)	10 (1)	1.80 ^c^	12.4	447.76	−11.5	0.7	0.938	300.87
4	105 (0)	1 (−1)	97.5 (0)	10 (1)	2.13 ^c^	11.8	380.58	−15.64	0.59	0.944	289.89
5	130 (1) ^b^	35.5 (0) ^b^	97.5 (0) ^b^	3 (−1) ^b^	2.87 ^c^	16.7	−	−	−	−	−
6	105 (0) ^a^	35.5 (0) ^a^	97.5 (0) ^a^	6.5 (0) ^a^	2.13 ^c^	13.3	370.60	−12.88	0.59	0.928	231.27
7	105 (0)	70 (1)	97.5 (0)	3 (−1)	2.13 ^c^	15.1	391.32	−7.89	0.62	0.933	253.21
8	130 (1) ^b^	35.5 (0) ^b^	97.5 (0) ^b^	10 (1) ^b^	2.87 ^c^	12.5	−	−	−	−	−
9	105 (0)	70 (1)	97.5 (0)	10 (1)	2.13 ^c^	10.7	461.80	−18.68	0.52	0.869	240.61
10	80 (−1)	35.5 (0)	97.5 (0)	10 (1)	1.40 ^c^	11.0	601.45	−12.18	0.66	0.945	394.76
11	130 (1) ^b^	70 (1) ^b^	97.5 (0) ^b^	6.5 (0) ^b^	2.87 ^c^	12.0	−	−	−	−	−
12	130 (1) ^b^	1 (−1) ^b^	97.5 (0) ^b^	6.5 (0) ^b^	2.87 ^c^	14.9	−	−	−	−	−
13	105 (0)	70 (1)	45 (−1)	6.5 (0)	1.80 ^c^	13.9	465.38	−8.09	0.67	0.937	292.96
14	130 (1) ^b^	35.5 (0) ^b^	45 (−1) ^b^	6.5 (0) ^b^	2.54 ^c^	13.8	−	−	−	−	−
15	105 (0)	1 (−1)	45 (−1)	6.5 (0)	1.80 ^c^	13.7	345.79	−11.72	0.58	0.941	243.89
16	105 (0)	1 (−1)	150 (1)	6.5 (0)	2.32 ^c^	13.3	375.02	−11.32	0.75	0.942	238.38
17	80 (−1)	1 (−1)	97.5 (0)	6.5 (0)	1.40 ^c^	11.1	503.23	−9.6	0.67	0.939	238.33
18	105 (0) ^a^	35.5 (0) ^a^	97.5 (0) ^a^	6.5 (0) ^a^	2.13 ^c^	13.1	400.36	−12.9	0.60	0.925	229.20
19	105 (0)	35.5 (0)	150 (1)	3 (−1)	2.32 ^c^	16.1	357.7	−9.2	0.74	0.942	223.80
20	80 (−1)	35.5 (0)	97.5 (0)	3 (−1)	1.40 ^c^	14.2	394.74	−6.2	0.57	0.899	193.95
21	105 (0)	1 (−1)	97.5 (0)	3 (−1)	2.14 ^c^	14.1	461.32	−8.33	0.69	0.942	280.59
22	130 (1) ^b^	35.5 (0) ^b^	150 (1) ^b^	6.5 (0) ^b^	3.06 ^c^	12.6	−	−	−	−	−
23	105 (0)	70 (1)	150 (1)	6.5 (0)	2.32 ^c^	11.5	225.30	−18.96	0.51	0.889	146.54
24	80 (−1)	37.5 (0)	45 (−1)	6.5 (0)	1.06 ^c^	12.8	374.60	−9.52	0.55	0.867	240.21
25	105 (0)	35.5 (0)	150 (1)	10 (1)	2.32 ^c^	10.0	232.12	−18.86	0.52	0.898	149.21
26	105 (0) ^a^	35.5 (0) ^a^	97.5 (0) ^a^	6.5 (0) ^a^	2.14 ^c^	13.1	400.11	−12.78	0.60	0.930	229.02
27	80 (−1)	35.5 (0)	150 (1)	6.5 (0)	1.59 ^c^	12.5	493.19	−15.6	0.68	0.930	309.95

Symbols (−1), (0), and (+1) represent the levels associated with each factor as considered in the construction of the Box-Behnken design used for the PHWE recovery process of the crude agar. ^a^ Represents the variables and responses found at the center points in the Box-Behnken design and allowed experimental replications. ^b^ The structure of the agar gel obtained did not withstand the double compression required to obtain the textural responses due to the unstable property of the gels recovered at highly severe PHWE treatments. ^c^ The severity factor was determined based on the combined effects of the operating temperature and recovery time of the PHWE process using an empirical equation (Equation (8)).

**Table 2:** ANOVA test of the gel hardness, gel adhesiveness, gel gumminess, and gel springiness parameters in relation to the variables of the pressurized hot water extraction process.

**Gel Hardness (g)**				**Gel Adhesiveness (g.s^−1^)**			
	**Mean Square**	**F Value**	***p*-Value**		**Mean Square**	**F Value**	***p*-Value**
Model	17,373.824	8.295	0.0006	Model	43.124	24.011	<0.0001
A-Temperature	20,907.702	9.982	0.0075	A-Temperature	16.124	8.978	0.0096
B-Pressure	904.972	0.432	0.5224	B-Pressure	4.679	2.605	0.1288
C-Time	34,978.803	16.700	0.0013	C-Time	60.319	33.585	<0.0001
D-Solid content	2748.371	1.312	0.2726	D-Solid content	134.616	74.952	<0.0001
AC	25,167.781	12.016	0.0042	BC	31.753	17.680	0.0009
AD	38,138.035	18.209	0.0009	CD	11.256	6.267	0.0253
BC	18,131.969	8.657	0.0114	Residual	1.796		
Residual	2094.482			Lack of Fit	2.056	8.608	0.1087
Lack of Fit	2422.068	8.273	0.1127	Pure Error	0.240		
Pure Error	292.760						
R-Squared (%)	82	Adjusted R-Squared (%)	72	R-Squared (%)	93	Adjusted R-Squared (%)	86
**Gel Gumminess (g)**				**Gel Springiness**			
	**Mean Square**	**F Value**	***p*-Value**		**Mean Square**	**F Value**	***p*-Value**
Model	6997.542	8.815	0.0004	Model	0.001	5.764	0.0033
A-Temperature	2811.539	3.542	0.0824	A-Temperature	0.000	1.662	0.2198
B-Pressure	1567.003	1.974	0.1835	B-Pressure	0.002	9.737	0.0081
C-Time	16,602.064	20.915	0.0005	C-Time	0.001	4.285	0.0589
D-Solid content	475.399	0.599	0.4528	D-Solid content	0.002	6.785	0.0218
AC	10,291.264	12.964	0.0032	AC	0.003	12.580	0.0036
AD	18,701.733	23.560	0.0003	AD	0.002	9.479	0.0088
BC	4963.907	6.253	0.0266	BD	0.001	4.713	0.0491
Residual	793.804			Residual	0.000		
Lack of Fit	926.937	15.053	0.0639	Lack of Fit	0.000	6.425	0.1423
Pure Error	61.577			Pure Error	0.000		
R-Squared (%)	82.5	Adjusted R-Squared (%)	73.2	R-Squared (%)	75.3	Adjusted R-Squared (%)	62

**Table 3:** Coded regression equations based on the polynomial two-factor interaction model of the different textural responses of the pressurized hot water extraction recovered agar gel from the algae industry waste stream.

Equation of Coded Values	
Gel hardness = 385.024 − 69.846A − 9.513B − 66.12375C−18.535D − 125.41275AC − 154.39AD − 67.3275BC	(10)
Gel cohesiveness = 0.62466667 + 0.02466667A − 0.03625B − 0.00625C − 0.0425D + 0.06375AB − 0.07125AC − 0.0875AD − 0.0825BC − 0.075CD	(11)
Gel gumminess = 242.608667 − 25.613A − 12.518B − 45.555C − 7.70875D − 80.2AC − 108.11375AD − 36.2275BC	(12)
Gel adhesiveness = −12.428 − 1.93966667A − 0.684B − 2.456C − 3.669D − 2.8175BC − 1.6775CD	(13)
Gel springiness = 0.9278 + 0.00946667A − 0.015B − 0.011125C − 0.014D − 0.042625AC − 0.037AD − 0.0165BD	(14)

## Data Availability

Data are contained within the article.

## Supplementary Materials

The following supporting information can be downloaded at: https://www.mdpi.com/article/10.3390/foods11142081/s1, Figure S1: Correlation of the predicted texture values from the regression model against the actual experimental values resulting from the pressurized hot water recovery of the crude agar gel from the algae waste stream; Figure S2: Response surface contour plots showing the combined effects of the pressurized hot water extraction (PHWE) parameters on the cohesiveness of the recovered crude agar gels; Figure S3: Response surface contour plots depicting the interrelated effects of the pressurized hot water extraction (PHWE) parameters on the gumminess of crude agar gel recovered from the algae industry waste stream; Figure S4: Response surface contour plots depicting the combined effects of pressurized hot water extraction (PHWE) parameters on the adhesiveness of the crude gel from the recovered residual agar; Figure S5: Three-dimensional response surface contour plots showing the combined effects of the pressurized hot water extraction (PHWE) parameters on the springiness of recovered crude agar gels; Table S1: Pearson Moment correlation showing the relationship between the severity factor, thermal hysteresis, texture, and physical properties of the recovered crude agar gel (1.5 wt.%). The Pearson coefficient (r) shows the direction of the relationship and the p-value evaluates the significance of the linear relationship at a 5% tolerance level; Table S2: Experimental values used in the determination of the partial (for each response) and combined multiresponse desirability tests.
