# Supplementary material for: Adjustable Gel Texture of Recovered Crude Agar Induced by Pressurized Hot Water Treatment of Gelidium sesquipedale Industry Waste Stream: An RSM Analysis
Source: Foods. 2022 Jul 13;11(14):2081. doi: 10.3390/foods11142081 (PMC9320704; doi:10.3390/foods11142081)
Supplement: Supplementary file 1 [file foods-11-02081-s001.zip › foods-1764748-supplementary.pdf]

Supplementary Materials

Adjustable gel texture of recovered crude agar induced by pressurized hot water treatment of *Gelidium sesquipedale* industry waste stream.

Cherif Ibrahima Khalil Diop [1,*], Sagrario Beltran [1], Isabel Jaime [2] and Maria-Teresa Sanz [1]

1 Chemical Engineering Section, Biotechnology and Food Science Department, University of Burgos, Pza. Misael Bañuelos s/n, 09001 Burgos, Spain; beltran@ubu.es (S.B.); tersanz@ubu.es (M.-T.S.)
2 Food Technology Section, Biotechnology and Food Science Department, University of Burgos, Pza. Misael Bañuelos s/n, 09001 Burgos, Spain; ijaime@ubu.es
* Correspondence: cidiop@ubu.es; Tel.: +34-633-308-654

Table S1. Pearson Moment correlation showing the relationship between the severity factor, thermal hysteresis, texture and physical properties of the recovered crude agar gel (1.5 wt.%). The Pearson coefficient (r) sowed the direction of the relationship and the p-value evaluated the significance of the linear relationship at 5% tolerance level.

		**Hysteresis (ºC)**	**Gel Hardness (g)**	**Gel Adhesiveness (g)**	**Severity Factor**	**Gel Springiness (mm)**	**Gel Cohesiveness**	**Gel Gumminess (g)**
Hysteresis (ºC)	r coeff.	-	0.4659	0.4257	-0.5088	0.2985	0.2274	0.4245
	P-value	-	0.0333	0.0543	0.0185	0.1887	0.3216	0.0551
Gel Hardness (g)	r coeff.	0.4659	-	0.308	-0.4646	0.4152	0.4235	0.878
	P-value	0.0333	-	0.1744	0.0339	0.0612	0.0557	0.000
Gel Adhesiveness ($g.s^{-1}$)	r coeff.	0.4257	0.308	-	-0.4222	0.3898	0.3786	0.257
	P-value	0.0543	0.1744	-	0.0566	0.0807	0.0905	0.2608
Severity Factor	r coeff.	-0.5088	-0.4646	-0.4222	-	0.1301	0.1476	-0.3659
	P-value	0.0185	0.0339	0.0566	-	0.574	0.5232	0.1028
Gel Springiness (mm)	r coeff.	0.2985	0.4152	0.3898	0.1301	-	0.6501	0.5182
	P-value	0.1887	0.0612	0.0807	0.574	-	0.0014	0.0161
Gel Cohesiveness	r coeff.	0.2274	0.4235	0.3786	0.1476	0.6501	-	0.4805
	P-value	0.3216	0.0557	0.0905	0.5232	0.0014	-	0.0275
Gel Gumminess (g)	r coeff.	0.4245	0.878	0.257	-0.3659	0.5182	0.4805	-
	P-value	0.0551	0.000	0.2608	0.1028	0.0161	0.0275	-

Table S2. Experimental values used in the determination of the partial (for each response) and combined multiresponse desirability tests.

Name	Goal	Lower Limit	Upper Limit	Weight	Importance
A: Temperature	Minimize	100[a]	130	1	4
B: Pressure	In range	1	70	1	3
C: Time	Minimize	45	150	1	4
D: Solid content	Maximize	3	10	1	3
Gel Hardness	Maximize	225.3	601.45	1	4
Gel Adhesiveness	Minimize	-18.96	-6.2	1	3
Gel Springiness	Maximize	0.867	0.945	1	3
Gel Cohesiveness	Maximize	0.47	0.75	1	3
Gel Gumminess	In range	146.54	394.76	1	3

a - The lower limit value of the Pressurized hot water temperature was deliberately fixed to 100 °C.

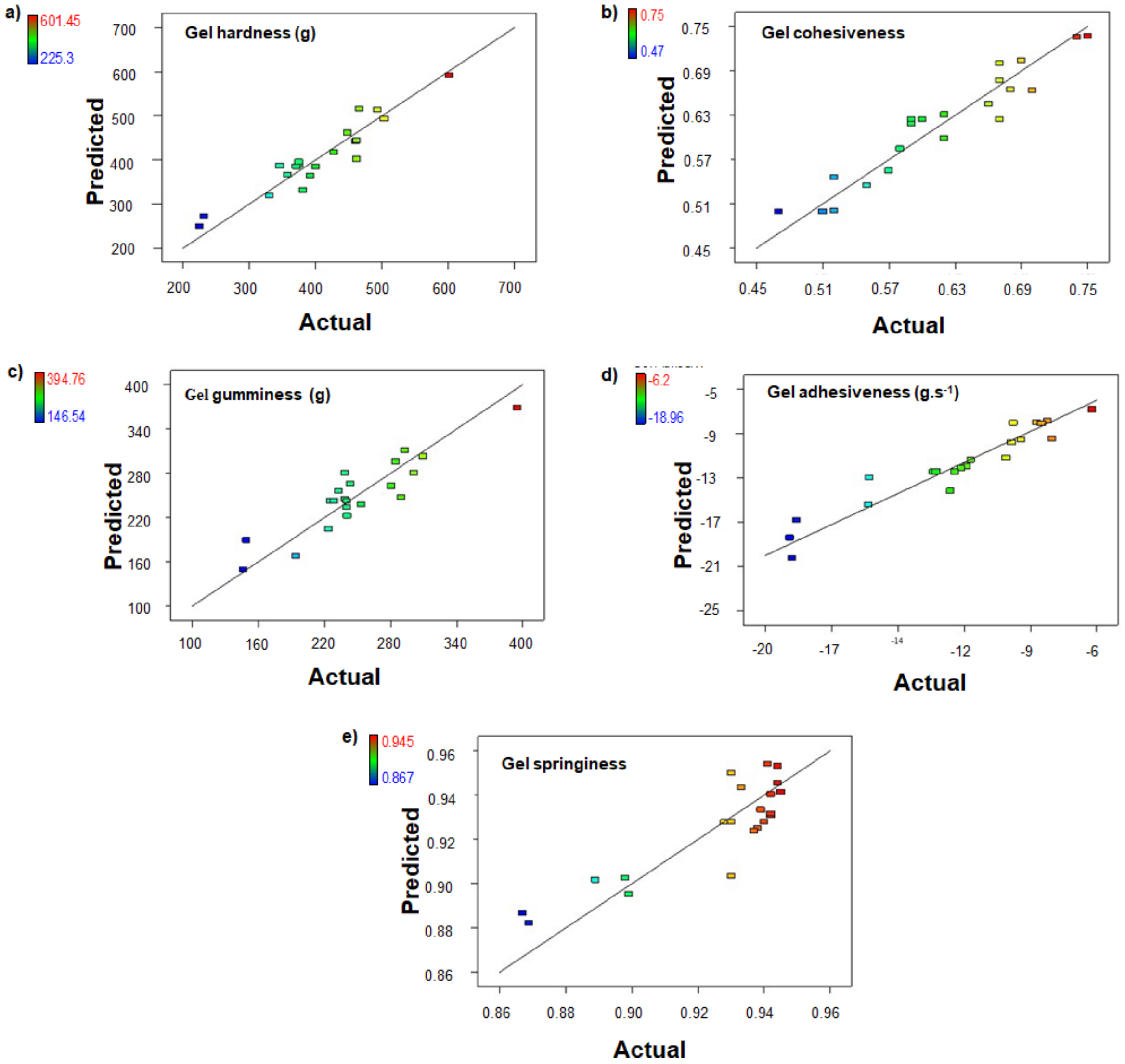

Figure S1. Correlation of the predicted texture values from the regression model against the actual experimental values resulting from the pressurized hot water recovery of the crude agar gel from the algae waste stream.

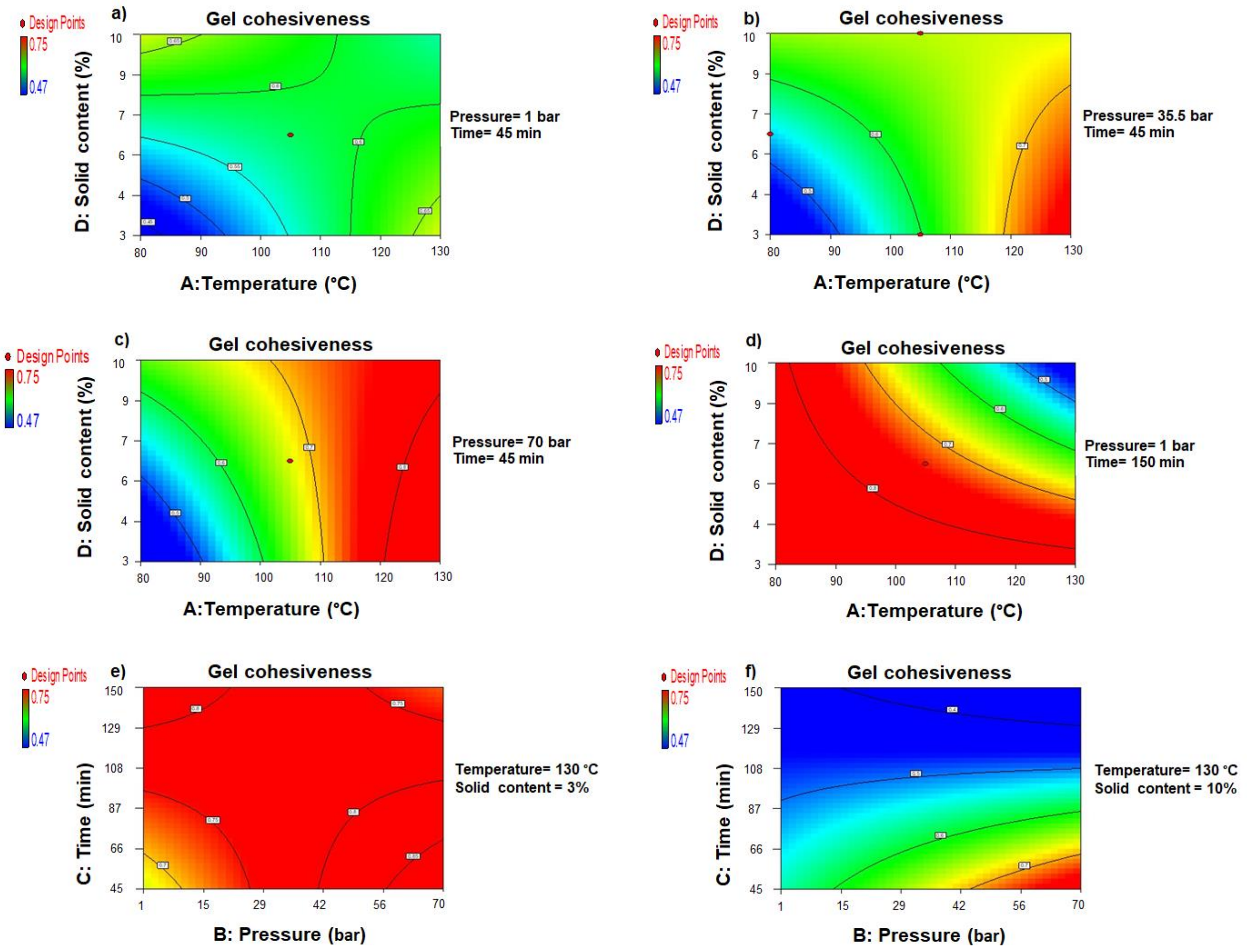

Figure S2. Response surface contour plots showing the combined effects of the pressurized hot water extraction (PHWE) parameters on the cohesiveness of the recovered crude agar gels.

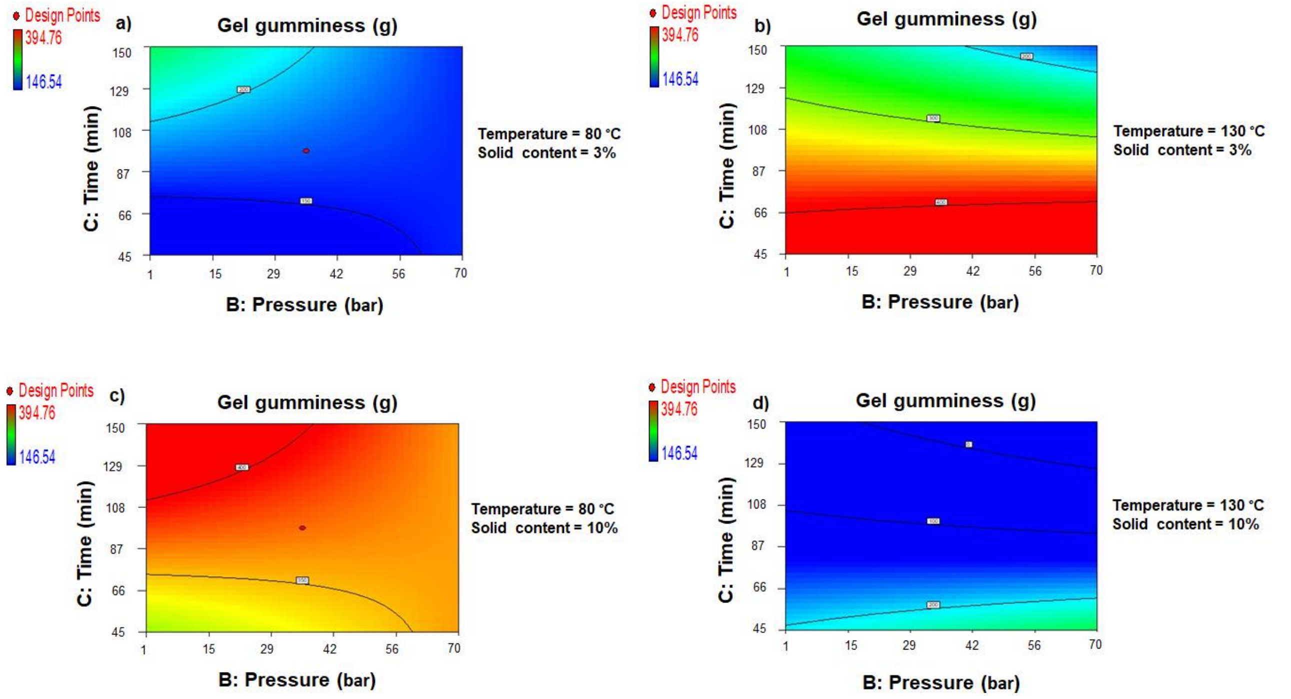

Figure S3. Response surface contour plots depicting the interrelated effects of the pressurized hot water extraction (PHWE) parameters on the gumminess of crude agar gel recovered from the algae industry waste stream.

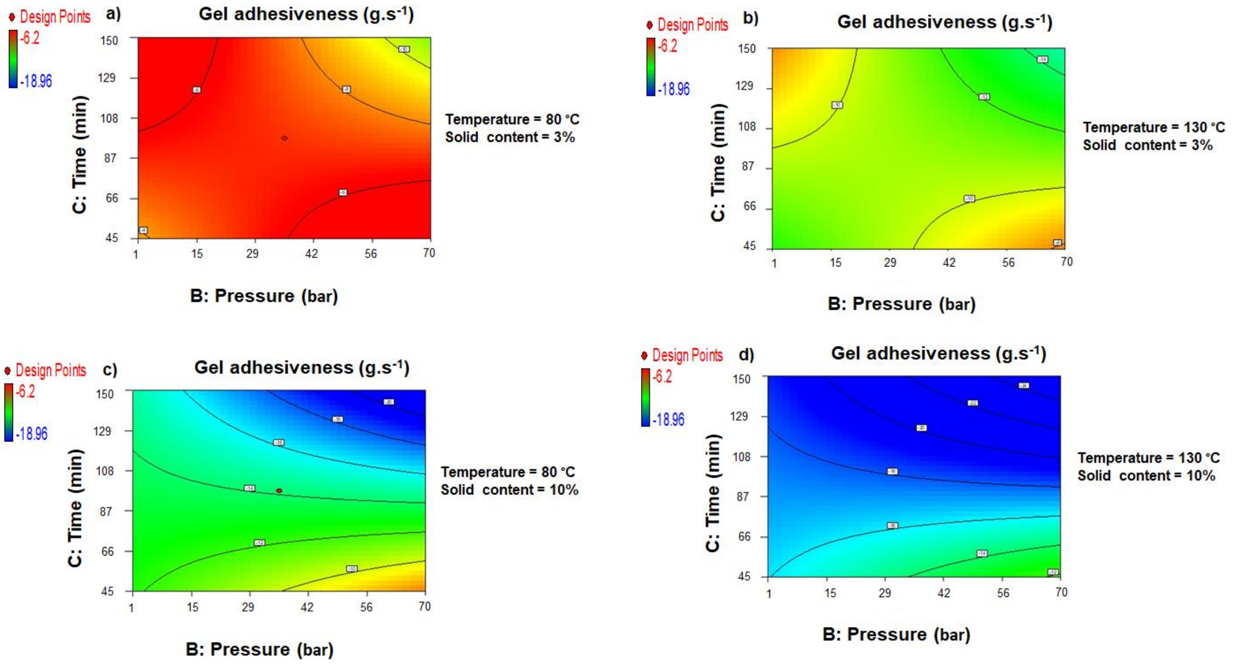

Figure S4. Response surface contour plots depicting the combined effects of pressurized hot water extraction (PHWE) parameters on the adhesiveness of the crude gel from the recovered residual agar.

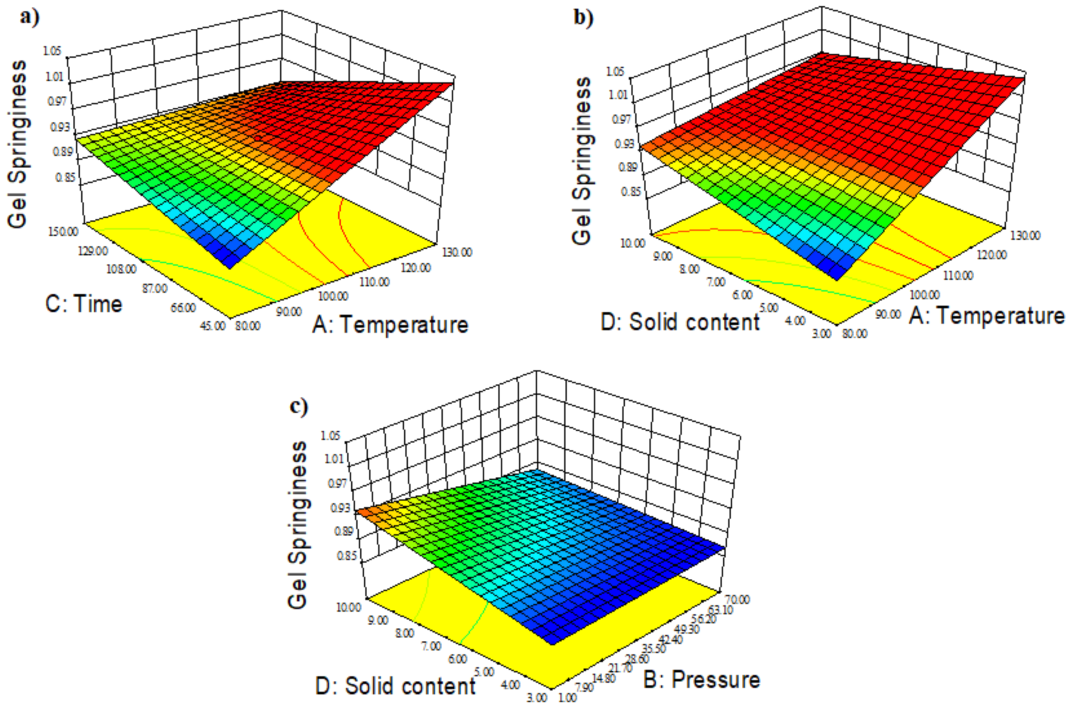

Figure S5. 3D Response surface contour plots showing the combined effects of the pressurized hot water extraction (PHWE) parameters on the springiness of recovered crude agar gels.
